# Impact of COVID-19 pandemic among patients with lung and head and neck cancer assisted in a public cancer center in Brazil

**DOI:** 10.1186/s12885-024-12255-0

**Published:** 2024-04-29

**Authors:** Gilson Gabriel Viana Veloso, Flávia Duarte Amaral, Angélica Nogueira-Rodrigues

**Affiliations:** 1 MD, Post-Graduate Program in Health Sciences, Faculdade Ciências Médicas de, Minas Gerais – FCM-MG, Oncologist, Oncoclínicas&Co/MedSir, Belo Horizonte, MG Brazil; 2 Oncoclínicas&Co/MedSir, Belo Horizonte, MG Brazil; 3 Post-Graduate Program in Health Sciences, Faculdade Ciências Médicas de Minas Gerais – FCM-MG, Federal University of Minas Gerais – UFMG, Brazilian Group of Gynecologic Oncology (EVA), Oncoclínicas&Co/MedSir, DOM Oncologia, Belo Horizonte, MG Brazil

**Keywords:** Cancer, COVID-19, Clinical oncology, Head and Neck tumors, Lung tumors

## Abstract

**Background:**

There is no updated national data regarding the real impact of the COVID-19 pandemic on delaying diagnosis and treatment among patients with lung, and head, and neck cancers in Brazil. This study aimed to analyze the COVID-19 pandemic impact on cancer diagnosis and clinical outcomes among lung, head, and neck cancer patients assisted in a tertiary cancer center in Southeastern Brazil, as well as to analyze these patients’ pretreatment clinical features.

**Methods:**

Retrospective cohort of patients with lung or head and neck cancer assisted in a tertiary cancer center in southeastern Brazil between January/2019 and December/2021. To assess statistical differences among groups [i.e., cohort 2019 versus (vs.) 2020 and 2019 vs. 2021] chi-square test was used with a 5% significance level and 90% power for sample size calculation. Differences among baseline clinical features and sociodemographic characteristics were evaluated either by T-test for two samples or Fisher’s or Pearson’s chi-square test (for quantitative or qualitative variables). All utilized tests had a 5% significance level.

**Results:**

Six hundred fifty-two patients were included, 332 with lung and 320 with head and neck cancer; it was observed a significant decrease in oncologic treatment recommendations and increase in palliative care recommendation for patients with lung cancer, despite similar stages at diagnosis. During the COVID-19 pandemic, more pain symptoms were reported at the first Oncology assessment for patients with head and neck cancer (*p* < 0.05). Compared to 2019, head and neck cancer patients diagnosed in 2021 presented a worse initial performance status (*p* = 0.008). There was a statistically significant increase in survival for patients diagnosed with head and neck cancer in 2021 when compared to 2019 (*p* = 0.003).

**Conclusions:**

This research highlights low survival rates for patients with lung and head and neck cancer in Brazil, even before the pandemic started, as consequence of advanced diseases at diagnosis at the public health system and clinical degrading features. Additionally, there was an increase incidence in both lung cancer and head and neck cancer despite no differences in clinical stage. This reflects how fragile is the public healthcare system even before facing an acute public health crisis such as the COVID-19 pandemic. Yet, the total impact on public health may follow for many years.

## Background

Cancer is now the first or second leading cause of death in over 60% of the countries, according to the World Health Organization (WHO) [1], and nearly 70% of cancer deaths occur in middle and low-income countries (LMICs) [2]. In 2020, 225,830 Brazilians died from the disease [3].

The Brazilian Ministry of Health estimates nearly 704,000 new cancer cases in the country for each year between 2023 and 2025 [3]. Regarding lung cancer, 32,560 new cases are expected per year, placing this tumor as the fourth most common cancer in the country [3]. Focusing on oral cavity and laryngeal cancers, nearly 15,100 and 7,790 new cases per year are expected, respectively [3].

Since the beginning of the coronavirus-19 pandemic (COVID-19), by the end of January 2023, over 6,7 million people have died from the disease worldwide, including nearly 700,000 Brazilians [4], and since its’ beginning, oncologists were concerned about its impact on patient care [5].

The overall distraction among health care systems due to COVID-19 may implicate deleterious effects on cancer patient assistance in the short and long-term follow-up [6] and quantifying the impact of delayed cancer diagnosis and treatment due to the pandemic in both the clinical stage and the prognosis is a complex task [7].

The main objective of this study was to analyze COVID-19 pandemic impact on cancer diagnosis among lung and head and neck cancer patients assisted in a tertiary cancer center in Southeastern Brazil as well as to analyze these patients’ pretreatment clinical features.

## Materials and methods

### Study protocol

This was a retrospective cohort study at *Santa Casa de Misericórdia de Belo Horizonte*, a tertiary cancer center in Belo Horizonte, Southeastern Brazil. This study aimed to analyze whether the COVID-19 pandemic harmed patients with lung and head and neck cancers, yielding delayed diagnosis, more advanced clinical stage at diagnosis, and poorer outcomes in comparison with 2019, the year before the pandemic. For that aim, three cohorts were defined including patients diagnosed between 2019 and 2021, one cohort for each year. To proper collect and access data, two separate comparisons were defined: 2019 vs. 2020 and 2019 vs. 2021. In 2020 Brazil had major changes concerning lockdowns, restructure in the hospital to become a respiratory hospital for COVID-19 patients and major impact on increased deaths. In 2021, Brazil’s scenario started to change with vaccination coverage in January 2021, flexibilization of lockdown regimens and hospital’s regaining the opportunity to fully work as they usually did before the pandemic has started (i.e., 2019).

### Population of analysis

Inclusion criteria included patients with a confirmed diagnosis of either head and neck cancer or lung cancer, above 18 years old, who had their first oncology assessment between January first, 2019, and December thirty first, 2021. Subjects could have had their first oncology assessment either by a hospital admission or on an ambulatory basis.

Exclusion criteria included: patients with inconclusive biopsies for malignant neoplasia, thyroid cancer, thymic tumors, and pleural mesothelioma. Those tumors were excluded from the final analysis due to different biological behavior in comparison to the other tumors here assessed.

Tumors were codified according to the International Classification of Diseases 10th edition (ICD-10) [8] as here described: C00, C01, C02, C03, C04, C05, C06, C07, C08, C09, C10, C11, C12, C13, C30, C31, C32, C33, C34, C43 and C80.

### Data collection

Data collection was performed through a structured questionnaire developed by the authors aiming to assess all proposed objectives in this research. All charts were available for consultation at the hospital’s electronic system for assistant physicians.

### Covariates

For lifestyle habits associated with head and neck cancer, the following variables were included in the questionnaire: smoking and alcohol intake histories, divided into 2 categories: current or former and never; and, and body mass index (BMI – kg/m²), analyzed continuously and then categorized based on cutoffs for malnutrition (< 18,5 kg/m²), adequate (18,5–24,9 kg/m²), overweight (≥ 25,0 kg/m²), and obesity (≥ 30,0 kg/m²).

Sociodemographic variables included race and education level. Race was categorized into four categories: undeclared, Caucasian, black, and mixed race. Education level was categorized into 2 categories: ≤ 8 years of formal education and > 8 years of formal education. Financial status was initially included as a sociodemographic variable, however, due to a lack of data on charts (100% of misinformation) it was excluded.

To assess patients’ clinical features the following variables were included: clinical or pathologic stage, performance status at first oncologic assessment and after 6 months, treatment indication (including all modalities, i.e., surgery, chemotherapy, and/or radiotherapy), the indication of exclusive palliative care at first oncology assessment, pain symptoms reported, necessity of enteral nutrition, the indication of tracheostomy, and dental work-up before treatment started. The latter was used solely for patients with head and neck cancer. A dichotomized strategy was used (i.e., yes, or no) for the necessity of enteral nutrition, the indication of treatment, the indication of exclusive palliative care at first oncology assessment, pain symptoms, the indication of tracheostomy, and dental work-up before treatment.

Patients were staged accordingly to the TNM Classification of Malignant Tumors 8th edition by the American Joint Committee on Cancer [9]. Performance statuses were assessed based on the Eastern Cooperative Oncology Group (ECOG) scale, divided into six categories (range 0 to 5) [10]. Outcome analysis and objective response rate were based on the guideline for Response Evaluation Criteria for Solid Tumors (RECIST) 1.1 [11].

Two other variables included in this research were the time between primary biopsy and first oncology assessment and the time between the first appointment with an oncologist and the date of the first treatment. Means and standard deviation were calculated.

Overall survival analysis was calculated based on the date of diagnosis and date of death registered on the chart or loss of follow-up. Cutoff date for overall survival was January 15th, 2023. For the cause of death, patients were stratified into death due to baseline disease, due to COVID-19 infection or due to other cause (yes or no categories for all). Additionally, a dichotomous variable “death before treatment start” was included for those patients with treatment indication but who died before its’ beginning, also categorized into “yes” or “no”.

### Statistical analysis

**Fig. 1:**
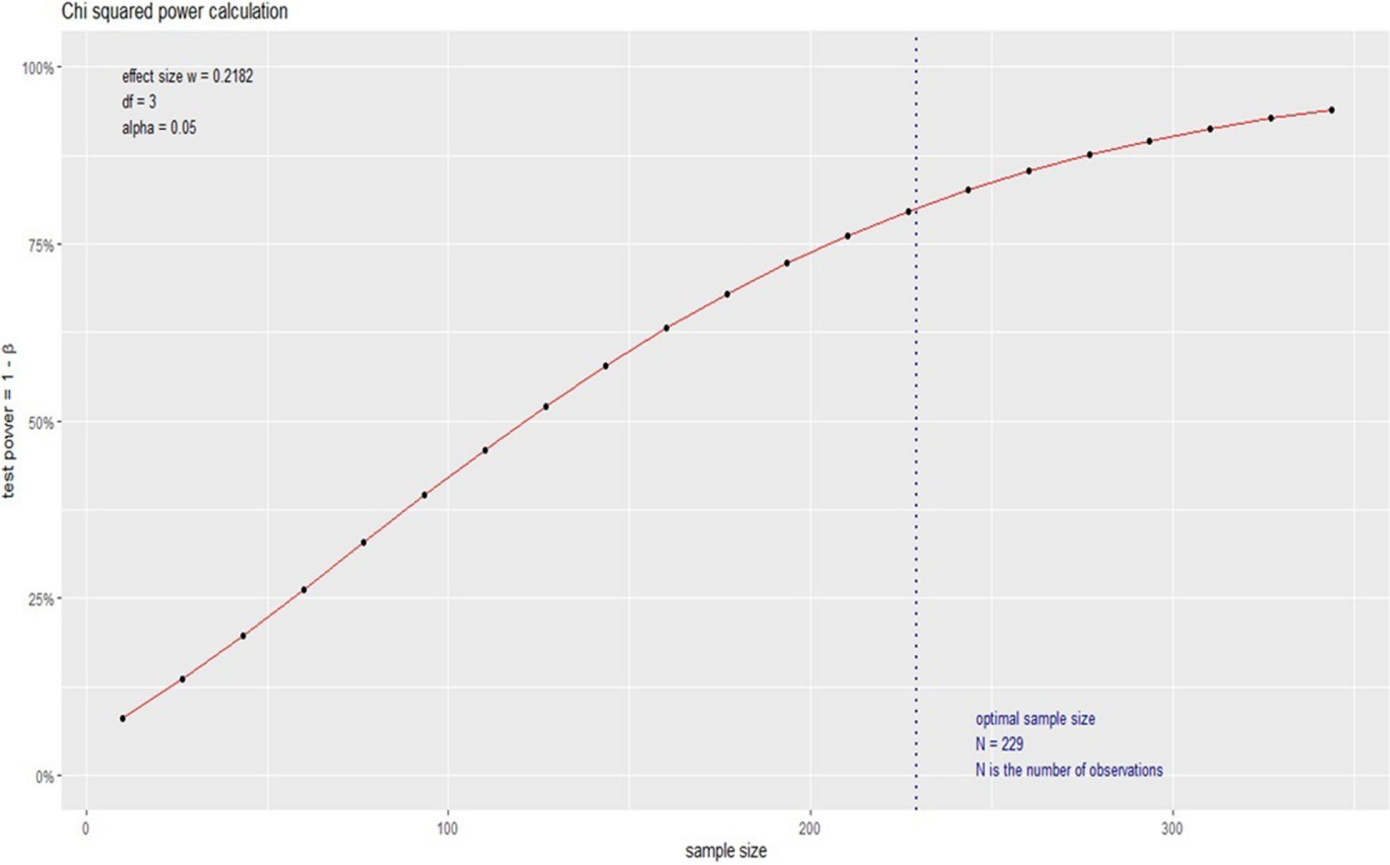
Sample size calculation

To assess statistical differences among groups [i.e., 2019 versus (vs.) 2020 and 2019 vs. 2021] a chi-square (χ²) test was used for two independent groups with a 5% significance level and 90% power for sample size calculation. To estimate measurement effectiveness a pilot study was performed by an independent statistician with 40 patients randomly selected from the patients described in Table 1. Based on these data, the sample size was calculated on R statistic software version 3.5.1 with an effect of 0,2182. The minimum sample size for our planned analysis was then 298 patients (Fig. 1).

Differences among baseline clinical features and sociodemographic characteristics were evaluated either by T-test for two samples or Fisher’s or Pearson’s chi-square test (for quantitative or qualitative variables). T-test for 2 samples was used based on the central limit theorem, which states that in sample sizes above 5 or 10 per group, all means present with normal distribution, independently of data distribution [12]. All utilized tests had a 5% significance level (meaning a *p*-value ≤ 0,05).

All statistical analyses were performed on IBM SPSS Statistics (SPSS, version 23.0 for Windows; SPSS Inc, Chicago, Ill).

### Ethics

This study was submitted and approved by the Ethics Committee of *Santa Casa de Misericórdia de Belo Horizonte* (approval number 39,115,720,900,005,138). Since this is a retrospective cohort and there was a substantial number of reported deaths on the medical charts with no possibility for verbal or written consent, the need for informed consent was waived by *Ethics Committee of Santa Casa de Misericórdia de Belo Horizonte*.

## Results

### Population characteristics

Six hundred fifty-two patients were included in the current analysis, 332 with lung cancer and 320 with head and neck cancer.

Focusing on the lung cancer patients, 87 patients were diagnosed in 2019, 124 in 2020 and 121 in 2021. In 2019 and 2020, the mean age at diagnosis was 66 years old, and 65 in 2021 (Table 1). In 2020, females were predominant; in 2019 and 2021 men were the majority. Baseline characteristics that showed a significant statistical difference among groups were smoking status (25% never smokers in 2020 compared to 10% in 2019, *p*-value 0.027) and race (25% of Caucasians in 2020 and 29% in 2021, compared to 17% in 2019, *p*-values 0.001 for both comparisons). Although non-significant, patients diagnosed during the pandemic presented with more pain symptoms (6% increase in 2020 and 12% increase in 2021, *p*-value 1.000 and 0.132, respectively) (Table 2). The time frame between first oncology assessment and initial treatment was shorter 20 days in 2020 compared to 2019 (*p*-value 0.029). For the time gap analysis, we included patients with treatment recommendations (i.e., 68 patients in 2019, and = 74 patients in 2020). There was no difference when it comes to clinical stage and metastatic disease at diagnosis to all comparisons for patients with lung cancer (2019 vs. 2020 and 2019 vs. 2021).

**Table 1:** Baseline characteristics of patients with lung cancer 2019-2021

	2019 *n* = 87	2020 *n* = 124	*p*-value 2019 vs. 2020	2021 *n* = 121	*p*-value 2019 vs. 2021
Age at start, mean (SD), y	66.52 (9.71)	66.67 (12.46)	0.924	65.33 (10.86)	0.418
Gender, No. (%)			0.328		0.052
Male	48 (55.2)	59 (47.6)		83 (68.6)	
Female	39 (44.8)	65 (52.4)		38 (31.4)	
Smoking status, No. (%)			0.027		0.122
Current or former	62 (87.3)	84 (73)		89 (77.4)	
Never	9 (12.7)	31 (27)		26 (22.6)	
Alcohol intake, No. (%)			0.594		0.590
Current or former	31 (62)	52 (55.9)		71 (67)	
Never	19 (38)	41 (44.1)		35 (33)	
Body mass index, mean (SD), kg/m²	22.35 (5.12)	22.72 (5.6)	0.636	22.57 (4.18)	0.758
Missing, No. (%)					
Body mass index, No. (%)			0.602		0.288
< 18.50	18 (25.7)	20 (19.8)		21 (22.6)	
18.50 – 24.90	33 (47.2)	51 (50.5)		43 (46.2)	
25.0 – 29.90	12 (17.1)	23 (22.8)		25 (26.9)	
> 30.0	7 (10)	7 (6.9)		4 (4.3)	
Education level, No. (%)			0.237		0.789
< 8 formal years of study	18 (78.3)	65 (74.7)		59 (73.8)	
≥ 8 formal years of study	5 (21.7)	22 (25.3)		21 (26.2)	
Race, No. (%)			0.001		0.001
Undeclared	12 (13.8)	1 (0.8)		4 (3.3)	
Caucasian	15 (17.2)	31 (25.2)		35 (28.9)	
Black	3 (3.4)	11 (8.9)		17 (14.0)	
Mixed race	57 (65.5)	80 (65)		65 (53.7)	
Necessity of enteral nutrition, No. (%)			1.000		0.579
Yes	7 (8.1)	10 (8.1)		7 (5.8)	
No	79 (90.8)	113 (90.8)		114 (94.2)	
First oncology assessment, No. (%)					
Hospital	41 (47.1)	68 (54.8)	0.327	65 (53.7)	0.399
Ambulatory	46 (52.9)	56 (45.2)		56 (46.3)	
Clinical stage (0 – IV), No. (%)			0.106		0.124
0	-	-		1 (0.8)	
I	-	3 (2.4)		1 (0.8)	
II	3 (3.4)	1 (0.8)		7 (5.8)	
III	21 (24.1)	20 (16.1)		14 (11.6)	
IV	63 (72.4)	100 (80.6)		98 (81.0)	
Stratified clinical stage, No. (%)			0.478		0.059
0	-	1 (0.8)		-	
IA 1-3	-	1 (0.8)		-	
IB	-	2 (1.6)		1 (0.8)	
IIA	2 (2.3)	-		2 (1.6)	
IIB	1 (1.1)	1 (0.8)		6 (5.1)	
IIIA	13 (14.9)	12 (9.8)		8 (6.8)	
IIIB	6 (6.9)	6 (4.8)		4 (3.1)	
IIIC	2 (2.3)	2 (1.6)		2 (1.6)	
IVA	36 (41.4)	51 (41.1)		41 (33.9)	
IVB	27 (31.0)	48 (38.7)		57 (47.1)	
Tumor size – T, No. (%)			0.604		0.775
Tx	7 (8.1)	9 (7.2)		1 (0.8)	
Tis	-	-		6 (5.0)	
T0	-	-		1 (0.8)	
T1	5 (5.7)	5 (4.1)		5 (4.1)	
T2	18 (20.7)	23 (18.5)		24 (19.8)	
T3	16 (18.4)	16 (12.9)		16 (13.2)	
T4	41 (47.1)	71 (57.3)		68 (56.2)	
Node status – N, No. (%)			0.908		0.822
Nx	5 (5.7)	4 (3.2)		11 (9.1)	
N0	35 (40.2)	55 (44.4)		42 (34.7)	
N1	15 (17.2)	20 (16.1)		19 (15.7)	
N2	24 (27.6)	34 (27.4)		35 (28.9) 14 (11.6)	
N3	8 (9.2)	11 (8.9)			
Metastatic disease – M1, No. (%)					
Yes	63 (72.4)	101 (81.5)	0.133	98 (81.0)	0.179
No	24 (27.6)	23 (18.5)		23 (19.0)	
Initial performance status – ECOG, No. (%)			0.669		0.484
0	4 (4.6)	14 (11.3)		19 (15.7)	
1	29 (33.3)	55 (44.4)		48 (39.6)	
2	13 (14.9)	20 (16.1)		24 (19.8)	
3-4	14 (9.2)	26 (8.9)		24 (8.9)	

*SD* Standard deviation, *ECOG* Eastern Cooperative Oncology Group

**Table 2:** Clinical features of patients with lung cancer 2019–2021

	2019 *n* = 87	2020 *n* = 124	*p*-value 2019 vs. 2020	2021 *n* = 121	*p*-value 2019 vs. 2021
Patient with treatment recommendation, No. (%) Yes No	77 (88.5) 10 (11.5)	95 (76.6) 29 (23.4)	0.031	88 (72.7) 33 (27.3)	0.006
Initial surgical treatment, No. (%) Yes No	5 (5.7) 82 (94.3)	7 (5.6) 117 (94.4)	1.000	4 (3.3) 117 (96.7)	0.496
Chemotherapy recommendation, No (%) Yes No	75 (86.2) 12 (13.8)	91 (73.4) 33 (26.6)	0.027	86 (71.1) 35 (28.9)	0.012
Radiotherapy recommendation, No. (%) Yes No	34 (39.1) 53 (60.9)	36 (29) 88 (71)	0.139	39 (32.2) 82 (67.8)	0.377
Indication of exclusive palliative care, No. (%) Yes No	8 (9.2) 79 (90.8)	28 (22.6) 96 (77.4)	0.015	33 (27.3) 88 (72.7)	0.001
Pain symptoms reported, No (%) Yes No	35 (48.6) 37 (51.4)	58 (47.5) 64 (52.5)	1.000	70 (60.3) 46 (39.7)	0.132
Indication of tracheostomy, No (%) Yes No	- 87 (100)	2 (1.6) 122 (98.4)	0.513	2 (1.6) 122 (98.4)	0.335
Time between biopsy result and first oncology assessment, mean (SD), days	87.80 (392.20)	28.40 (31.08)	0.094	28.01 (33.35)	0.096
Time between first oncology assessment and initial treatment, mean (SD), days	73.43 (64.22)	53.15 (44.45)	0.029	74.38 (66.84)	0.933

*SD* Standard deviation

Regarding the group with head and neck cancer, the mean age at diagnosis was 55.49 in 2019, 58.50 years in 2021, and 60.05 years in 2021 (Table 3); the latter with a statistically significant difference (*p*-value 0.012). Males were predominant in all three years. The sole baseline characteristic that showed a significant statistical difference among groups was race (25% Caucasians in 2020 compared to 17% in 2019, and 28.9% of Caucasians in 2021 compared to 17% in 2019; *p*-value 0.000 for both comparisons). There was a 22% increase in primary tumor size (tumors classified as “T4”) among patients with head and neck cancer in 2020 in comparison to 2019 (*p*-value 0.017). Presence of pain symptoms had nearly a 11% increase in 2020, and a 18% increase in 2021 when compared to 2019 (*p*-value 0.002 and 0.029, respectively) (Table 4). In 2021, for the initial performance status, there was a 11% increase in category “2” (*p*-value 0.008). Also, in 2021 the indication of tracheostomy had a 15% increase in comparison to 2019 (*p*-value 0.043). For patients in the head and cancer group, there was no difference when it comes to clinical stage and metastatic disease at diagnosis to all comparisons (2019 vs. 2020 and 2019 vs. 2021). However, there was a tendency to the increased clinical stage in 2021 (*p*-value 0.058 for the stratified clinical stage).

**Table 3:** Baseline characteristics of patients with head and neck cancer 2019–2021

	2019 *n* = 82	2020 *n* = 125	*p*-value 2019 vs. 2020	2021 *n* = 113	*p*-value 2019 vs. 2021
Age at start, mean (SD), y	55.49 (13.71)	58.50 (11.59)	0.091	60.05 (11.38)	0.012
Gender, No. (%)					
Male	69 (84.1)	99 (79.2)	0.468	93 (82.3)	0.847
Female	13 (15.9)	26 (20.8)		20 (17.7)	
Smoking status, No. (%)					
Current or former	62 (86.1)	115 (95)	0.055	102 (91.9)	0.224
Never	10 (13.9)	6 (5)		9 (8.1)	
Alcohol intake, No. (%)					
Current or former	58 (82.9)	101 (85.6)	0.678	98 (90.7)	0.161
Never	12 (17.1)	17 (14.4)		10 (9.3)	
Body mass index, mean (SD), kg/m²					
Missing, No. (%)	20.21 (4.78)	20.59 (4.62)	0.582	20.54 (4.63)	0.646
Body mass index, No. (%)					
< 18.50	32 (41)	45 (38.5)	0.270	37 (36.6)	0.604
18.50 – 24.90	31 (39.7)	53 (45.3)		45 (44.6)	
25.0 – 29.90	14 (17.9)	13 (11.1)		15 (14.9)	
> 30.0	1 (1.4)	6 (5.1)		4 (3.9)	
Education level, No. (%)					
< 8 formal years of study	29 (76.3)	84 (88.4)	0.106	78 (82.1)	0.473
≥ 8 formal years of study	9 (23.7)	11 (11.6)		17 (17.9)	
Race, No. (%)					
Undeclared	10 (12.2)	1 (0.8)	0.005	2 (1.8)	0.002
Caucasian	12 (14.6)	22 (17.6)		27 (23.9)	
Black	14 (17.1)	27 (21.6)		33 (29.2)	
Mixed race	46 (56.1)	75 (65)		51 (45.1)	
Necessity of enteral nutrition, No. (%)					
Yes	45 (56.2)	75 (60)	0.663	53 (46.9)	0.243
No	35 (43.8)	50 (40)		60 (53.1)	
First oncology assessment, No. (%)					
Hospital	24 (29.3)	43 (34.4)	0.453	31 (27.4)	0.872
Ambulatory	50 (70.7)	82 (65.6)		82 (72.6)	
Clinical stage (0 – IV), No. (%)					
0	-	1 (0.8)	0.529	-	0.842
I	3 (3.7)	5 (4)		2 (1.8)	
II	4 (4.9)	5 (4)		6 (5.3)	
III	13 (15.9)	11 (8.8)		16 (14.2)	
IV	62 (75.6)	103 (82.4)		89 (78.8)	
Stratified clinical stage, No. (%)					
0	-	1 (0.8)	0.202	-	0.058
I	3 (3.8)	5 (4.1)		2 (1.8)	
II	4 (5.1)	5 (4.1)		6 (5.4)	
III	13 (16.6)	11 (9.1)		16 (14.9)	
IV A	42 (53.8)	54 (45.8)		38 (35.5)	
IV B	10 (13.5)	33 (27)		30 (28.4)	
IV C	6 (7.2)	11 (9.1)		15 (14.0)	
Tumor size – T, No. (%)					
Tx	4 (4.9)	5 (4.0)		6 (5.3)	
Tis	-	1 (0.8)		-	
T0	-	-		-	
T1	4 (4.9)	6 (4.8)		4 (3.5)	
T2	10 (12.2)	9 (7.2)		11 (9.7)	
T3	23 (28.0)	14 (11.2)		31 (27.4)	
T4	41 (50.0)	90 (72.0)	0.017	61 (54.0)	0.927
Node status – N, No. (%)					
Nx	4 (4.9)	5 (4.0)	0.417	6 (5.3)	0.118
N0	26 (31.7)	32 (25.6)		29 (25.7)	
N1	9 (11.0)	21 (16.8)		25 (22.1)	
N2	34 (41.5)	47 (37.6)		33 (29.2)	
N3	9 (11.0)	20 (16.0)		20 (17.7)	
Metastatic disease – M1, No. (%)					
Yes	10 (12.2)	16 (12.8)	0.898	21 (18.6)	0.242
No	72 (87.8)	109 (87.2)		92 (81.4)	
Tumor primary site – ICD-10, No. (%)					
Oral cavity (ICD-10 C00-C06; C09)	27 (32.9)	48 (38.4)	0.809	18 (15.9)	0.116
Salivary glands (ICD-10 C07-C08)	2 (2.4)	2 (2.4)		3 (2.7)	
Oropharynx (ICD-10 C10)	19 (23.2)	28 (22.4)		33 (28.3)	
Nasopharynx (ICD-10 C11)	3 (3.7)	5 (4.0)		6 (5.3)	
Larynx (ICD-10 C32)	12 (14.6)	23 (18.4)		33 (28.3)	
Hypopharynx (ICD-10 C13)	11 (13.4)	12 (14.6)		12 (10.6)	
Nasal cavity/middle ear (ICD-10 C30)	1 (1.2)	-		-	
Accessory sinuses (ICD-10 C31)	3 (3.7)	1 (1.2)		2 (1.8)	
Primary unknown (ICD-10 C80)	4 (4.9)	5 (4.0)		6 (5.3)	
Malignant melanoma (ICD-10 C43)	-	1 (1.2)		-	
Initial performance status – ECOG, No. (%)					
0	14 (17.7)	23 (18.4)	0.078	31 (29)	0.008
1	45 (57)	58 (46.4)		48 (44.9)	
2	6 (7.6)	26 (20.8)		20 (18.7)	
3-4	14 (17.7)	18 (14.4)		8 (7.5)	

*Abbreviation*: *SD* standard deviation, *ICD-10* International Classification of Diseases 10th edition, *ECOG* Eastern Cooperative Oncology Group

**Table 4:** Clinical features of patients with head and neck cancer 2019–2021

	2019 *n* = 82	2020 *n* = 125	*p*-value 2019 vs. 2020	2021 *n* = 113	*p*-value 2019 vs. 2021
Patient with treatment recommendation, No. (%) Yes No	78 (95.1) 4 (4.9)	114 (91.2) 11 (8.8)	0.431	101 (89.4) 12 (10.6)	0.190
Initial surgical treatment, No. (%) Yes No	24 (29.3) 58 (70.7)	33 (26.4) 92 (73.6)	0.751	28 (24.8) 85 (75.2)	0.515
Chemotherapy recommendation, No (%) Yes No	67 (81.7) 15 (18.3)	94 (75.2) 31 (24.8)	0.308	90 (79.6) 23 (20.4)	0.855
Radiotherapy recommendation, No. (%) Yes No	74 (90.2) 8 (9.8)	105 (84.0) 20 (16.0)	0.220	98 (86.7) 15 (13.3)	0.507
Indication of exclusive palliative care, No. (%) Yes No	5 (6.1) 77 (93.9)	13 (10.4) 112 (89.6)	0.324	11 (9.7) 102 (90.3)	0.435
Pain symptoms reported, No (%) Yes No	30 (56.6) 23 (43.4)	84 (67.2) 22 (17.2)	0.002	80 (74.8) 27 (25.2)	0.029
Indication of tracheostomy, No (%) Yes No	32 (39.0) 50 (61.0)	62 (50.4) 61 (49.6)	0.118	61 (54.0) 52 (46.0)	0.043
Dental work-up before treatment, No (%) Yes No	20 (25.0) 62 (75.0)	37 (31.4) 81 (68.6)	0.343	28 (27.2) 75 (72.8)	0.866
Time between biopsy result and first oncology assessment, mean (SD), days	74.65 (71.58)	62.24 (77.40)	0.247	64,64 (92.30)	0.414
Time between first oncology assessment and initial treatment, mean (SD), days	52.41 (54.38)	41.51 (27.49)	0.111	58.25 (66.32)	0.573

*SD* Standard deviation

### Outcomes and survival analysis

Regarding treatment recommendation for patients with lung cancer, including chemotherapy indication, it was observed a 12% decrease in treatment recommendations in 2020 compared to 2019 (*p*-value 0.031), and 16% decrease in 2021 (*p*-value 0.006), irrespectively of curative intention (i.e., first-line treatment also). Moreover, there was a 11% increase in the indication of exclusive palliate care at first oncology assessment in 2020 (*p*-value 0.015) and 18% in 2021 (*p*-value 0.001). All comparisons were with patients diagnosed in 2019, the year before the pandemic.

**Fig. 2:**
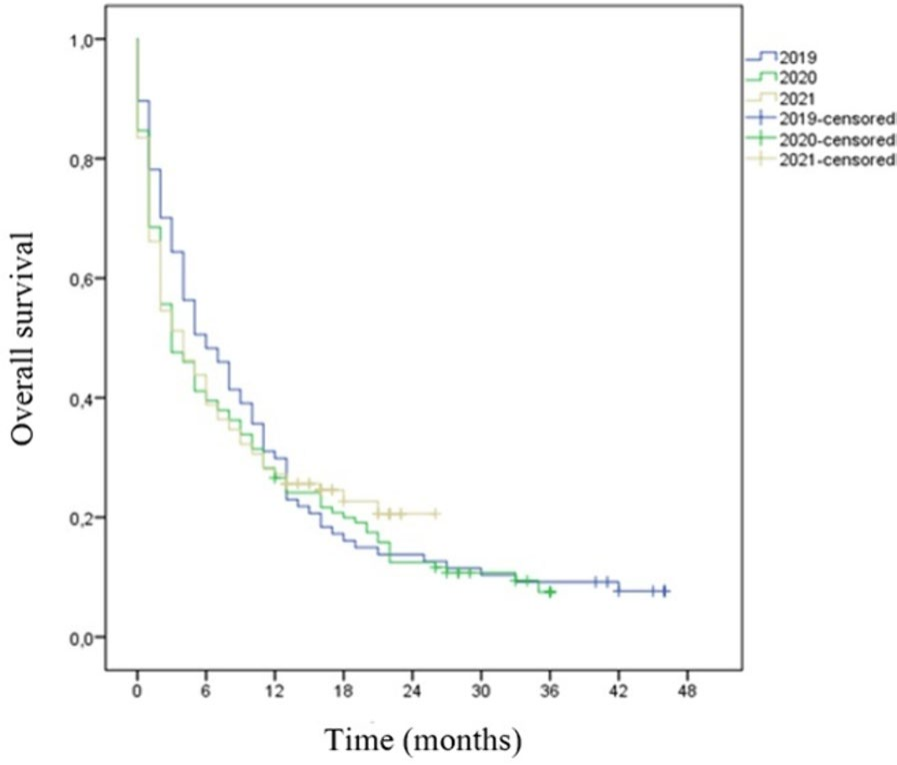
– Overall survival for patients with lung cancer, 2019–2021

There were no statistically significant differences between patients’ outcomes for lung cancer when patients from 2019 to 2020 were compared (Table 5). For overall survival there was a non-significant reduction in 2020 survival [6 months (95% CI 3.18–8.81 months) in 2019 vs. 3 months in 2020 (95% CI 1.18–4.81)] (Table 5; Fig. 2). There was no statistically significant difference in objective rate response among patients with lung cancer in 2019 vs. 2021 group. Performance status after 6 months of first oncology assessment showed an increase in death rate after 6 months for 2021’s patients (*p* = 0.001). Even though there was a statistically significant difference regarding survival rate (*p* = 0.005), overall survival showed a non-significant 33% decrease in 2021 survival [6 months (95% CI 3.18–8.81 months) vs. 4 months (95% CI 2.41–5.59)] (Table 5; Fig. 2).

**Table 5:** Baseline characteristics of patients with lung cancer 2019-2021

	2019 *n* = 87	2020 *n* = 124	*p*-value 2019 vs. 2020	2021 n = 121	*p*-value 2019 vs. 2021
Death, No. (%)					
Yes	80 (92)	112 (90.3)	0.809	93 (76.9)	0.005
No	7 (8.0)	12 (9.7)		28 (23.1)	
Death due to baseline disease, No. (%)					
Yes	75 (86.2)	104 (83.9)	0.844	86 (71.1)	0.012
No	12 (13.8)	20 (16.1)		35 (28.9)	
Death before treatment start, No. (%)					
Yes	7 (8.0)	16 (12.9)	0.270	17 (14)	0.196
No	80 (92)	108 (87.1)		104 (86)	
Death due to COVID-19 infection, No. (%)					
Yes	4 (4.6)	6 (4.9)	1.000	4 (3.3)	0.722
No	83 (95.4)	118 (95.1)		117 (96.7)	
Primary outcome after 6 months, No. (%)					
Complete response	4 (4.7)	7 (5.6)	0.746	2 (1.7)	0.129
Partial response	11 (12.9)	11 (8.9)		15 (12.4)	
Stable disease	7 (8.2)	10 (8.1)		17 (14.0)	
Disease progression	16 (18.8)	18 (14.5)		11 (9.1)	
Death	47 (58.8)	78 (62.9)		76 (62.8)	
	-	-		-	
Performance status after 6 months – ECOG, No. (%)					
0	2 (2.5)	11 (9.0)	0.189	12 (10)	0.001
1	12 (15)	16 (13.1)		26 (21.7)	
2	9 (11.2)	8 (6.6)		4 (3.3)	
3-4	10 (12.5)	9 (7.4)		2 (1.7)	
5	47 (58.8)	78 (63.9)		76 (63.3)	
Objective rate response (%)	15 (17.2)	18 (14.5)	0.544	17 (13.7)	0.549
Overall survival, median (SD), months	6.0 (1.43)	3,0 (0.97)	0.733*	4.0 (0.81)	0.733^a^

*Abbreviation*: *SD* Standard deviation, *ECOG* Eastern Cooperative Oncology Group

^a^Result of log-rank test for overall survival

**Fig. 3:**
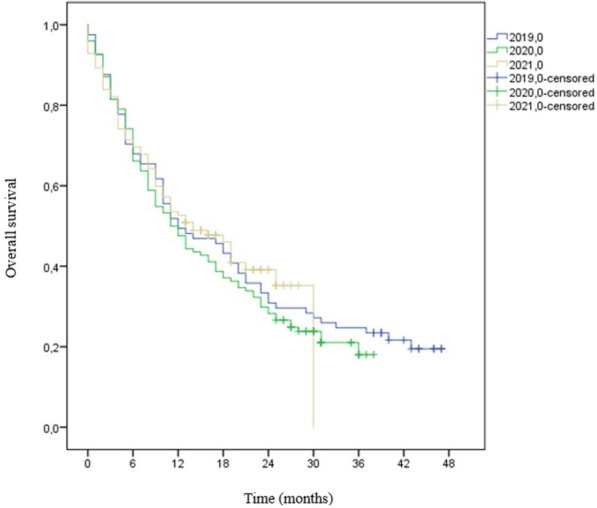
– Overall survival for patients with head and neck cancer, 2019–2021

There were no statistically significant differences between patients’ outcomes for head and neck cancer when patients from 2019 to 2020 were compared (Table 6). There was a statistically significant difference in survival rate in 2019 vs. 2021 group for patients with head and neck cancer, with a 20% decrease for patients from 2021 (*p*-value 0.003). Additionally, performance status after 6 months of first oncology assessment showed an improvement after treatment with a 20% increase among patients categorized as “0” (*p* = 0.013). Differently from the lung cancer cohort, it was not observed a significant decrease in treatment recommendation or an increase in palliative care recommendation during the pandemic in the head and neck cancer cohort. Kaplan Meier’s curve for overall survival for head and neck cancer to all three years is demonstrated in Fig. 3.

**Table 6:** Outcomes: patients with head and neck cancer 2019–2021

	2019 *n* = 82	2020 *n* = 125	*p*-value	2021 *n* = 113	*p*-value 2019 vs. 2021
Death, No. (%) Yes No	65 (79.3) 17 (20.7)	98 (78.4) 26 (21.6)	1.000	66 (58.4) 47 (41.6)	0.003
Death due to baseline disease, No. (%) Yes No	63 (76.8) 19 (23.2)	90 (72) 35 (28)	0.518	64 (56.6) 49 (43.4)	0.004
Death before treatment start, No. (%) Yes No	11 (13.4) 71 (86.6)	19 (15.2) 106 (84.8)	0.841	13 (11.5) 100 (88.5)	0.826
Death due to COVID-19 infection, No. (%) Yes No	- 82 (100)	2 123 (98.4)	0.522	1 (0.9) 112 (99.1)	1.000
Primary outcome after 6 months, No. (%) Complete response Partial response Stable disease Disease progression Death	26 (31.7) 11 (13.4) 7 (8.5) 4 (4.9) 34 (41.5) -	35 (28.5) 14 (11.4) 6 (4.9) 18 (14.6) 50 (40.7) -	0.218	38 (33.9) 16 (14.3) 3 (2.7) 11 (9.8) 44 (39.3) -	0.308
Performance status after 6 months – ECOG, No. (%) 0 1 2 3–4 5	10 (12.3) 31 (38.3) 3 (3.7) 3 (3.7) 34 (42)	15 (12.4) 34 (28.1) 14 (11.6) 8 (6.6) 50 (41.3)	0.207	37 (33) 25 (22.3) 2 (1.8) 4 (3.6) 44 (39.3)	0.010
Objective rate response, (%)	45.12	35.8	0.471	55.5	0.772
Overall survival, median (SE), months	12.0 (3.6)	11.0 (1.5)	0.554*	14.0 (2.6)	0,554^*^

*Abbreviation*: *SE* Standard error, *ECOG* Eastern Cooperative Oncology Group

^*^Result of log-rank test for overall survival

## Discussion

The *Santa Casa de Misericórdia de Belo Horizonte* is a hospital with 1000 beds destined for public health care and is one of the biggest cancer centers in Minas Gerais state. The hospital is responsible for one-third of all oncology treatments on the providence. In 2019 24,666 oncology appointments, including patients for first assessment (hospital or ambulatory basis), patients in current anti-cancer treatment, patients in exclusive palliative care, and patients on follow-up after cancer remission. For 2020, the number of appointments were 24,547 and in 2021, 32,850. In addition, the Oncology service did not interrupt treatment assistance for cancer patients receiving chemotherapy and/or radiotherapy due to the pandemic. However, many surgeries were canceled due lack of beds in intensive care units (ICU) for post-operation observance since the ICU beds were relocated to patients with confirmed COVID-19 or acute respiratory distress. Also, during the COVID-19 pandemic, over 70% of these 1000 beds were turned into respiratory wings; such approach was also observed in other cancer centers in the country [13]. Worldwide the COVID-19 pandemic impacted cancer care; a global collaborative study across 54 countries [14] reported that in over 88% of the participating cancer centers there was challenges in providing adequate cancer care during the pandemic, including number of medical appointments, restricted access to medications, and missing on chemotherapy cycles. However, in this cancer center we were able to maintain the oncology wing in its’ full capacity and were able to increase the number of patients assisted.

Brazil is a middle-income country with over 207 million inhabitants (according to the 2022 census) [15]. Several low and middle-income countries are not prepared to provide adequate care to cancer patients, one of the reasons why high-income countries have higher survival rates [1]. The pillars of Brazil’s public health policies include equity, equality, and integrity to whoever seeks medical care in public health centers and Brazil’s Brazilian public health system provides free treatment to over 190 million people [16], including all cancer treatment modalities (i.e., surgery, chemotherapy, and radiotherapy) [17]. Since all these treatment options are fully funded by Brazilian’s government, patients’ assistance is completely free of charges, so, the resources offered to each patient is the same. Nonetheless, it is worth mentioning that the system may work with an important waiting list of medical appointments, diagnostic assessment, and treatment itself. Based on that, a Brazilian oncology patient loses nearly double of health years in comparison to some European countries and triple the time when compared to the United States of America [18]. Another fact that may impact overall cancer survival in Brazil is the difference between public health care and private care since there are several disparities among treatment options for locally advanced and advance diseases among these two groups. All these variables combined yield in low survival rate among cancer patients as it was demonstrated among the subjects included in this research.

Brazil has two specific laws regarding cancer treatment; the first one from 2012, known as the “60-day law” meaning that cancer patients have an upper limit of 60 days to initiate specific cancer treatment after diagnosis. The second one from 2019 is known as the “30-day law” meaning that highly suspicious patients have 30 days to fulfill all necessary diagnostic tests after symptoms have been reported to a medical physician [17]. Based on that, it was decided to assess among the subjects the time gap between the first oncology assessment and initial treatment; it was reported that in 2019 and 2021, the estimated time was close to 74 days, and for 2020, the estimated time was 53 days. National data indicates that most Brazilians receive a cancer diagnosis in the metastatic stage, reaching a peak of 200 days between the first reported symptom and biopsy release [17].

In this cohort, lung cancer patients diagnosed after the pandemic started shad a higher probability of not receiving chemotherapy and had a higher indication of exclusive palliative care, even though there was not a significant difference in clinical stage at diagnosis. To better define indication of best supportive care usually there’s a combination of ECOG scale plus functionality and nutritional status, and it was observed that patients were much more fragile than the pre-pandemic era and for our surprise, this scenario was irrespectively of clinical stage. Differences in treatment indication have not been observed in the head and neck cohort, but higher pain level at diagnosis was also observed.

In the lung cancer cohort 17.7% of patients had malnutrition, as for 35.6% in the head and neck cancer cohort. Brazilian nutritional status was updated in 2019, and results showed that 63% were above weight (overweight or obesity), 34.5% were eutrophic, and 2.5% were considered malnutrition [19]. In comparison to this study population, these updates showed that cancer patients may present with worst nutritional status than the general population, which is expected since involuntary weight loss is one of the first cancer symptoms. Moreover, patients with head and neck cancer may already experience reduced food intake before treatment starts [20–22], and patients with malnutrition have a higher risk of poor prognosis and worst treatment outcomes [23]. In this cohort, over 50% of patients with head and neck cancer patients needed enteral nutrition during treatment and that one-third suffered from malnutrition. Several aspects of the patient with head and cancer may alter due to malnutrition, such as impaired immune function, decrease in quality of life and interrupted treatments [20]. Pain tumor-related is an additional factor in weight loss [22], and we presented data with an increase in pain symptoms reported at diagnosis. The combination of our data based on nutritional status, and decreased functionality due to pain symptoms reinforce the findings of increased indication for exclusive palliative care it was found in this research.

Overall, pain symptoms were reported by 54.7% of the study population; 38% of patients in 2019 had pain symptoms reported. This number increased to 57% in 2020 and escalated to 31% in 2021. Here it must be highlighted that among patients with head and neck cancer, pain symptoms significantly increased after the COVID-19 pandemic started, whereas for lung cancer patients the reported increase was statistically non-significant. Since pain is one of the most challenging clinical features in cancer patients, it must be identified correctly and properly treated. Nearly 51% of all cancer patients report pain symptoms at some point during the disease (diagnosis, treatment, or exclusive palliative care) [24]. Patients with pain symptoms and delay medical care tend to seek self-medication, which may enhance treatment and clinical complications secondly to the mistreatment of their condition [25]. Lung cancer patients often report pain symptoms at diagnosis due to anatomic features of the tumor, such as bone and nerve invasion or metastatic disease, while patients with head and neck cancer may experience pain either due to the primary tumor or due to the treatment consequences, including surgery, radiation, or chemotherapy [24].

There was an increased number of lung cancer diagnoses on the hospital during the pandemic. For the lung cancer cohort, in 2020 there was an increase of 42.5% and in 2021 an increase of 39.1%. Concepcion et al. [26] reported an increase of 2.9% in 2020 and 3.34% in 2021 in total lung cancers diagnosed after the pandemic started but in much lower scale than it was found in this study. However, differently from what it was reported here, they showed a decrease in lung cancer death reports (-4.87% in 2020 and − 7.56% in 2021). Even though it was found an increased number of cancer diagnoses, Brazil had a decrease in such aspects during the pandemic, ranging from − 24.3% to -42.7% in some regions [27]; overall, up to 15,000 new cases were not diagnosed monthly due to COVID-19 [27]. Such data inference that oncology care varied in Brazilian territory during the pandemic – mainly due to lockdown recommendations and closed ambulatory services. Also, in contrast to these findings, a decreased incidence of lung cancer was observed by Kasymjanova et al. [28], with 34.7% less diagnosis but with more advanced stages during the COVID-19 pandemic. Regarding starting treatment with chemotherapy and/or radiotherapy, there was no significant delay.

Overall, there was no identification of statistical differences for the clinical stage at the lung cancer group, and it is worth mentioning that over 70% of subjects were metastatic at diagnosis [29]. The results presented by Lou et al. [30] also demonstrated no change in clinical stage at diagnosis for patients with lung cancer besides a shorter time-to-treatment in 2020 (38.92 days), like what it was found in this research. No change in clinical stage at diagnosis was also presented by Kizilirmak et al. [31]; stage IV disease was present in 59.31% of the pre-pandemic group and 65.35% of the pandemic group. Even though they did not find differences in lung cancer incidence between 2019 and 2020, Park et al. [32] identified a higher proportion of patients with locally advanced or metastatic disease after the COVID-19 pandemic started (2020 74.7% vs. 2109 62.7%).

Brazil does not provide thorax computerized tomography scans regularly due to high costs to the public health care system. There is no screening program for lung cancer in the country approved by Brazilian’s Ministry of Health, which might explain why Brazil has such high numbers of metastatic disease at diagnosis on a public health basis, which later will reflect in poorer outcomes since delayed diagnosis of lung cancer results in upper staging, decreased prognosis and lower survival rates [33]. Lung cancer has a 22.9% combined survival rate in five years [34], and clinical stage has an important role on these statistics, since clinical stage I ranges in survival rate from 92–68% in 5 years whereas patients with metastatic disease at diagnosis have a five-year survival rate of 10% [35].

Regarding the patients with head and neck cancer, there was an increase of 52.43% in 2020 and 37.8% in 2021. Nonetheless, Solis et al. [36] showed a 5–10% decrease in the number of new patients diagnosed with head and neck cancer after the COVID-19 pandemic, while several other international reports have documented a 22–43% decrease in the number of new diagnoses. Also, it was demonstrated patients with more advanced diseases when primary tumor size in 2020 was evaluated. In addition to the data here presented, the increased number of patients with tracheostomy indication may be related to such delayed diagnosis. Similarly, Tevetoğlu et al. [37], Flynn et al. [38], and Popovic et al. [39] also presented a cohort with patients presenting increased clinical stage at diagnosis among patients with head and neck cancer after the pandemic started. Although lymph node status is an important prognostic factor for these patients [40], this study did not find significant differences among all compared groups to this variable. The subgroup from 2021 did not showed differences among clinical stage, there was a tendency of more advanced diseased on the stratified staging. Similar results were presented by Clements et al. [41] who also did not find differences on symptoms and patients’ ECOG. The 5-year survival rates for head and neck cancer patients in general have a poor prognosis. The five-year survival rate varies between 30 and 70%, depending on the stage and location of the tumor [42]. In 2020 this cohort demonstrated a decreased survival in comparison to the pre-pandemic period. Similar results were cited by Peacok et al. [43]. As for 2021, there was a 20% increase in survival in comparison to 2019, but such significant difference may be due to a shorter follow-up in comparison to those patients from 2019, and perhaps such difference will balance after a 5-year follow-up.

In Brazil, public cancer centers receive a monthly amount of approximately $200,00 (R$ 1,100 Brazilian reais) from the government to treat patients with advanced lung cancer. For head and neck cancer the monthly amount of money range between $100,00 and $235,00 (R$ 571,00–1,300 Brazilian reais), irrespectively of clinical stage. The Brazilian healthcare system does not afford immunotherapy or direct target therapies for these tumors, except for gefitinib in patients with epidermal growth factor receptor (EGFR) mutations. In this context, that’s why Brazilian patients with lung, head and, neck cancer have access only to cytotoxic chemotherapy in contrast to all major recommendations for treatment worldwide [44, 45]. While lung cancer survival improved tremendously over the past 15 years since precision medicine arose, the main goal for thoracic oncologists is to overcome the median overall survival of 8 months that chemotherapy usually achieves [46]. Over 85% of patients with lung cancer included in this study died. For patients with head and neck cancer, over 71% have passed. These data put in evidence the disparities when adequate treatment access is not available, setting back the recent advances in modern oncology.

The data collected had an expressive amount of missing data for baseline characteristics, especially for 2019’s patients, with special attention to sociodemographic features, which may have increased the statistical differences among groups in those aspects. That is one of the pitfalls that follow retrospective studies; such differences in exposure data among groups may alter the study estimates [47].

In conclusion, in a cohort of 652 lung and head and neck cancer patients treated in Brazil from 2019 to 2021, it was observed a significant decrease in oncologic treatment recommendations and increase in palliative care indication during the first two years of the pandemic in the lung cancer group, despite similar stages at diagnosis. Increased pain levels at diagnosis were observed in all patients during the pandemic compared to patients diagnosed at the year before it. This study also highlights low survival rates for patients with lung and head and neck cancer in Brazil, even before the pandemic, as a probable consequence of advanced diseases at diagnosis and limited access to best treatment options at the publica health system.

## Data Availability

All data generated or analyzed during this study are included in this published article.

## Abbreviations

BMI: Body mass index
CI: Confidence interval
COVID: Coronavirus disease
ECOG: Eastern Cooperative Oncology Group
FCM-MG: Faculdades Ciências Médicas de Minas Gerais
GLOBOCAN: Global Cancer Statistics
ICD: International Classification of Diseases
ICU: Intensive Care Unit
LMICs: Low-and middle-income countries
RECIST: Response evaluation criteria in solid tumors
SD: Standard deviation
SE: Standard error
SEER: Surveillance, epidemiology, and end results program
TNM: Classification of tumors
VS: Versus
WHO: World Health Organization
